# Impact of dietary compositions and patterns on the prevalence of nonalcoholic fatty liver disease in Japanese men: a cross-sectional study

**DOI:** 10.1186/s12876-021-01919-x

**Published:** 2021-09-04

**Authors:** Chihiro Nakashita, Lu Xi, Yasushi Inoue, Ryota Kabura, Shota Masuda, Yuko Yamano, Takahiko Katoh

**Affiliations:** 1 Department of Public Health, Faculty of Life Sciences, Kumamoto University, 1-1-1 Honjou, Chuo-ku, Kumamoto, 860-8556 Japan; 2 Division of Food and Health Environmental Sciences, Faculty of Environmental and Symbiotic Sciences, Prefectural University of Kumamoto, 3-1-100 Tsukide, Higashi-ku, Kumamoto, 862-8502 Japan; 3 Department of Hygiene and Preventive Medicine, Showa University School of Medicine, 1-5-8 Hatanodai, Shinagawa-ku, Tokyo, 142-8555 Japan

**Keywords:** Nonalcoholic fatty liver disease, Diet, Liver, Obesity, Food consumption, Washoku

## Abstract

**Background:**

This study aimed to examine the impact of dietary compositions and patterns on nonalcoholic fatty liver disease (NAFLD) morbidity in Japanese men.

**Methods:**

We conducted a cross-sectional study of 281 individuals who underwent comprehensive medical examinations during health screening. Dietary intake was assessed using a semi-quantitative food frequency questionnaire, and factor analysis was performed to detect dietary patterns. NAFLD was diagnosed by the presence of fatty liver on abdominal ultrasonography in nondrinkers (< 30 g/day), and patients were categorized into control (n = 192) and NAFLD groups (n = 89).

**Results:**

Compared with the control group, the NAFLD group consumed fewer mushrooms. Three dietary patterns were identified, namely, a healthy pattern, a western pattern, and a snack pattern. The score of healthy pattern was negatively correlated with the risk of NAFLD. Compared with the lowest tertile of the healthy pattern, the middle tertile was associated with a lower risk of NAFLD after adjusting for age, physical activity, and smoking (odds ratio: 0.47, 95% confidence interval: 0.25–0.91). After further adjustments for body mass index, the middle tertile was associated with a lower risk of NAFLD (odds ratio: 0.46, 95% confidence interval: 0.23–0.92).

**Conclusions:**

A healthy dietary pattern comprising frequent intake of seaweeds, vegetables, mushrooms, pulses, and potatoes and starches was associated with a lower risk of NAFLD in Japanese men. In our opinion, this healthy pattern closely resembles the Japanese Washoku diet, indicating that adherence to Washoku may help prevent NAFLD.

## Background

Nonalcoholic fatty liver disease (NAFLD) includes several forms of liver diseases, ranging from simple steatosis to nonalcoholic steatohepatitis that can progress to cirrhosis and eventually to hepatocellular carcinoma. NAFLD is regarded as a hepatic phenotype of the metabolic syndrome, and obesity is a well-known major risk factor for NAFLD [1]. Accordingly, increase in the overweight population has caused NAFLD to become one of the most common liver diseases worldwide. Several recent studies estimated the association of NAFLD with economic burden. The latter is expected to increase as the prevalence of obesity and diabetes increases [2, 3]. NAFLD is prevalent in approximately 25% of the global population and in around 10% to 30% of the Japanese population [4–6].

Given that obesity is primarily related to dietary composition, several studies have clarified the relationship between diet and NAFLD. Previous studies have shown that lower intake of nutrient-rich and healthy foods (such as foods containing vitamin C, folate, and calcium, as well as vegetables, fruits, dairy products, nuts, and seeds) is associated with a significantly higher risk of developing NAFLD [7, 8]. Currently, dietary intake is mainly evaluated using dietary pattern analysis as this realistically assesses the overall dietary intake of single nutrients and the total dietary impact. Recent studies on NAFLD were also conducted using the dietary pattern analysis [9, 10].

Dietary patterns vary widely among countries. In Japan, the traditional diet is called Washoku. In 2013, the Washoku diet was added to the list of intangible cultural heritages by the United Nations Educational, Scientific and Cultural Organization. “One soup and three dishes” is the basic style of the Washoku diet. Specifically, this style is composed of the dietary staple (rice), soup, and several side dishes [11]. A large variety of foods (especially vegetables, fish, fruits, seaweed, and soybean foods) are consumed because diverse dishes comprise this diet [12]. Moreover, taking small portion sizes of various foods contributes to satiety and helps people to avoid overeating [13]. Thus, the Washoku diet has been noted to be a healthy dietary pattern owing to its low-calorie and well-balanced components [13].

However, despite this healthy diet, the prevalence of NAFLD remains high in Japan. One of the reasons may be the continuous westernization of diet, which is confirmed in Japan [14]. To the best of our knowledge, no study has investigated the association between dietary patterns and NAFLD in Japan. Therefore, this study aimed to investigate the association between dietary patterns and the risk of NAFLD in Japanese men.

## Methods

### Study design and participants

The participants were 992 Japanese men who underwent comprehensive medical examinations for health screening, including dietary intake assessment. The study was conducted at the Mitsui Memorial Hospital (Tokyo, Japan) between August 2005 and July 2006. Dietary intake was assessed using a food frequency questionnaire (FFQ). Participants with missing data on body measurements, blood biochemical composition, blood pressure, medical history, lifestyle, and dietary intake were excluded. Patients with chronic liver disease and hepatic carcinoma, those positive for the hepatitis B or C virus, or those who consumed > 30 g/day ethanol equivalent of alcohol were also excluded. Ultimately, 281 participants were included in the analysis. They were categorized into two groups, according to the presence of NAFLD: the control group (n = 192) and the NAFLD (n = 89) group.

### Clinical and laboratory assessments

All participants underwent an anthropometric assessment, as well as laboratory and radiological tests using standard methods. Information obtained included body mass index (BMI), body fat percentage, aspartate aminotransferase (AST), alanine aminotransferase (ALT), gamma-glutamyl transpeptidase (γ-GTP), total cholesterol (T-CHO), high-density lipoprotein cholesterol (HDL-C), low-density lipoprotein cholesterol (LDL-C), triglyceride (TG), systolic and diastolic blood pressure (SBP, DBP), fasting blood sugar (FBS), and hemoglobin A1c (HbA1c). Liver function was evaluated according to levels of high AST, ALT, γ-GTP, T-CHO, LDL-C, and TG in addition to the level of low HDL-C. Medical history, including hypertension and diabetes, and lifestyle, including physical activity and smoking behavior, were assessed using a self-administered questionnaire at the health screening. Participants’ levels of total physical activity were evaluated by determining the metabolic equivalent (MET) hours/day (METs score), which was calculated by multiplying the time spent on activities (/day) by MET intensity: walking slowly (2.0), walking quickly (4.0), light to moderate exercise (5.0), and strenuous exercise (7.0) for leisure-time physical activity [15]. The participants’ smoking behavior was categorized as follows: non-smoker, past smoker, and current smoker.

### Assessment of dietary intake and patterns

Food and beverage consumption were assessed using a validated semi-quantitative FFQ [16, 17] that comprised 138 food and beverage items, and usual consumption of the listed foods during the past year was also evaluated. Frequency responses consisted of nine options: less than once per month, 1–3 times per month, 1–2 times per week, 3–4 times per week, 5–6 times per week, once per day, 2–3 times per day, 4–6 times per day, and 7 or more times per day. Further, standard portion sizes were assessed for each food item with three choices: medium (standard), small (50% or less), and large (not less than 150%). Factor analysis (i.e., the principal factor method) was then performed to determine participants’ dietary patterns from 20 food groups in the FFQ. The factors identified were rotated using promax rotation. Eigenvalues and scree plots were used to decide which factors remained. Only foods with a factor loading ≥|0.35| were included in this study. The dietary patterns were classified on the basis of interpretation of foods with high factor loadings for each dietary pattern. The factor scores for each dietary pattern were calculated for each of the participants by summing intakes of food items weighted by their factor loadings. We also categorized the participants as per adherence to each dietary pattern (based on the tertiles of the dietary pattern scores) into low, medium, and high adherence groups.

### Assessment of fatty liver

Fatty liver was diagnosed using abdominal ultrasonography by experienced medical technologists or physicians, blinded to any relevant clinical information. NAFLD was diagnosed by the presence of fatty liver in nondrinkers (i.e., < 30 g/day) [18]. The median values (interquartile range [IQR]) of the ethanol equivalent of alcohol were 9.4 (0–20.0) and 0 (0–15.7) in the control and NAFLD groups, respectively.

### Statistical analysis

For clinical and dietary data, between-group comparisons were performed using the Mann–Whitney test for continuous variables and the chi-square test for categorical variables. Associations between the dietary compositions or patterns and NAFLD were estimated from odds ratios (ORs) and 95% confidence intervals (CIs) using multiple logistic regression analysis. Dietary patterns were generated using factor analysis. Energy and nutrient intakes by tertile of dietary patterns were compared using the Kruskal–Wallis test. All statistical analyses were performed using SPSS version 22 (IBM Corp., Armonk, NY, USA). *P* < 0.05 was considered to indicate statistical significance. The minimum number of participants required was determined by power analysis (A post hoc power analysis, G*power, Version 3.1.9.2 and sample size calculation for logistic regression [19]). A total of 281 participants were included in the data analysis.

## Results

Compared with the control group, the NAFLD group showed higher BMI, body fat percentage, SBP, DBP, FBS, and HbA1c, as well as worse liver function. However, there were no differences in levels of physical activity and total energy intake between the two groups. The clinical characteristics of the control and the NAFLD groups are shown in Table 1.

**Table 1:** Clinical characteristics of the subjects

Variables	Control (n = 192)	NAFLD (n = 89)	*P* value
Age (years)	61 (56–67)	62 (57–67)	0.751
BMI (kg/m^2^)	23.4 (21.8–24.8)	24.9 (23.7–26.5)	< 0.001
Body fat percentage (%)	20.9 (18.1–23.9)	24.4 (22.1–26.6)	< 0.001
SBP (mmHg)	130 (116–141)	137 (121–150)	0.014
DBP (mmHg)	81 (74–89)	86 (77–95)	0.03
FBS (mg/dl)	94 (89–102)	102 (96–114)	< 0.001
HbA1c (%)	5.2 (5.0–5.5)	5.5 (5.2–5.8)	< 0.001
AST (IU/L)	20 (17–23)	23 (19–30)	< 0.001
ALT (IU/L)	19 (15–25)	27 (21–41)	< 0.001
γ-GTP (IU/L)	31 (21–41)	38 (30–55)	< 0.001
T-CHO (mg/dl)	208 (188–227)	212 (194–231)	0.119
HDL-C (mg/dl)	52.6 (45.7–61.1)	47.7 (42.1–54.0)	< 0.001
LDL-C (mg/dl)	132 (112–149)	140 (124–159)	0.006
TG (mg/dl)	103 (74–146)	140 (102–184)	< 0.001
Physical activity (Ex)	1.86 (0.54–3.82)	1.50 (0.43–4.25)	0.262
Energy (kcal)	1664 (1374–1979)	1653 (1369–1950)	0.779
Hypertension n (%)	44 (22.9)	20 (22.5)	0.934
Diabetes n (%)	12 (6.3)	4 (4.5)	0.555
Smoking behavior n (%)			
Non smoker	79 (41.1)	24 (27.0)	0.022
Past smoker	87 (45.3)	46 (51.7)	0.320
Current smoker	26 (13.5)	19 (21.3)	0.097

Data are shown as the median (IQR) or numbers and percentages

*P* values were obtained from Mann–Whitney test (for continuous variables) and the chi-square test (for categorical variables)

Table 2 shows the dietary intake by food group as assessed using the FFQ. The NAFLD group consumed significantly fewer mushrooms (control: 6.5 (3.8–11.7) g/day; NAFLD: 4.4 (3.0–6.4) g/day; *P* < 0.01). There were no significant differences in relation to the other food group.

**Table 2:** Comparison of food group intakes according to food frequency questionnaire in the NAFLD and control subjects

	Control (n = 192)	NAFLD (n = 89)	*P* value
Cereals (g/day)	446.3 (389.3–527.2)	447.2 (395.7–523.7)	0.792
Potatoes and starches (g/day)	12.1 (7.1–19.1)	10.3 (7.1–16.9)	0.316
Sugars and starches (g/day)	4.0 (1.4–8.1)	4.3 (1.8–8.1)	0.503
Confectioneries (g/day)	15.2 (7.9–25.2)	17.7 (8.3–26.2)	0.626
Fats and oils (g/day)	9.2 (7.2–12.1)	8.9 (7.1–11.6)	0.580
Nuts and seeds (g/day)	1.2 (0.2–2.2)	0.9 (0.1–2.1)	0.268
Pulses (g/day)	56.5 (34.3–85.1)	57.3 (32.9–94.3)	0.891
Fishes and shellfishes (g/day)	64.3 (50.9–90.4)	65.3 (49.8–94.6)	0.906
Meat (g/day)	50.0 (33.5–68.0)	52.9 (38.1–67.3)	0.271
Eggs (g/day)	16.8 (10.1–34.1)	21.7 (12.4–31.7)	0.260
Milk and dairy products (g/day)	167.1 (95.4–283.2)	175.1 (110.3–262.0)	0.967
Vegetables (g/day)	134.2 (94.1–191.8)	112.7 (76.2–192.1)	0.178
Green and yellow vegetables (g/day)	65.5 (42.0–103.6)	56.1 (36.6–96.7)	0.144
Other vegetables (g/day)	61.6 (40.8–90.4)	55.7 (37.1–86.4)	0.457
Pickled vegetables (g/day)	9.8 (5.1–17.6)	12.1 (6.3–19.7)	0.241
Fruits (g/day)	172.8 (101.1–228.2)	164.7 (98.7–204.2)	0.273
Mushrooms (g/day)	6.5 (3.8–11.7)	4.4 (3.0–6.4)	0.001
Seaweeds (g/day)	6.4 (3.7–11.0)	5.9 (3.2–9.9)	0.468
Seasonings and spices (g/day)	7.7 (5.0–11.6)	6.3 (4.8–9.7)	0.139
Luxury beverages (g/day)	640.2 (431.1–889.2)	680.9 (509.3–909.6)	0.540

Data are shown as the median (IQR). *P* values were obtained from Mann–Whitney test

Adjusted for energy by using residual method

To investigate the dose-dependent effect of mushrooms, we classified participants into four groups according to the quartile of mushrooms intake. The multivariate OR value for mushrooms intake is shown in Table 3. Compared to the highest quartile (Q4), mushroom intake in the lowest quartile (Q1) was significantly associated with a higher risk of NAFLD after adjusting for age, physical activity, and BMI (OR: 3.36, 95% CI: 1.47–7.66). As mushroom intake decreases (Q2, Q1), the inverse association between mushroom consumption and NAFLD was increased.

**Table 3:** Multivariable analysis for the risk of NAFLD by quartile of mushrooms

Intake of mushrooms (g/day)	Crude OR (95%CI)	Multivariate-adjusted OR (95%CI)
Q4 (10.69 ≦)	1.0	1.0
Q3 (5.81–10.69)	1.63 (0.75–3.56)	2.17 (0.93–5.09)
Q2 (3.31–5.81)	2.41 (1.13–5.14)*	3.10 (1.35–7.11)*
Q1 (< 3.31)	2.88 (1.36–6.12)*	3.36 (1.47–7.66)*

Adjusted for age, physical activity, and BMI

Participants are classified four groups equally in the order of intakes

*p < 0.05

Three dietary patterns were identified by factor analysis, namely a healthy dietary pattern, a western dietary pattern, and a snack pattern (Table 4). The healthy dietary pattern was characterized by frequent intake of seaweeds, vegetables, mushrooms, pulses, and potatoes and starches. The western dietary pattern comprised fats and oils, meat, and seasonings and spices. The snack dietary pattern comprised sugars and starches, luxury beverages (tea, coffee, fruit juice, soft drinks, among other drinks.), and fruits. The proportion of total variance explained by the three dietary patterns was 37.8%.

**Table 4:** Factor-loading matrix for dietary patterns

	Dietary pattern		
	Healthy	Western	Snack
Seaweeds	0.67	–	–
Other vegetables	0.66	–	–
Mushrooms	0.60	–	–
Green and yellow vegetables	0.50	–	–
Pulses	0.49	–	–
Potatoes and starches	0.45	–	–
Pickled vegetables	0.39	–	–
Fats and oils	–	0.88	–
Meat	–	0.68	–
Seasonings and spices	–	0.52	–
Sugars and starches	–	–	0.72
Luxury beverages	–	–	0.56
Fruits	–	–	0.36
% of variance explained (%)	25.2	6.6	6.0

Absolute values < 0.3 were not presented for simplicity

Food items with factor loadings less than ± 0.3 for all dietary patterns (cereals, confectioneries, nuts and seeds, fishes and shellfishes, eggs, milk and dairy products, alcoholic beverages) were omitted

Table 5 shows the associations between dietary patterns and the risk of NAFLD. The score of healthy pattern was negatively correlated with NAFLD. As compared with the lowest tertile of the healthy pattern, the middle tertile was associated with a lower risk of NAFLD after adjusting for age, physical activity, and smoking (OR: 0.47, 95% CI: 0.25–0.91). After further adjustments for BMI, the middle tertile was associated with a lower risk of NAFLD (OR: 0.46, 95% CI: 0.23–0.92). However, the highest tertile of the healthy pattern showed no significant association. There was also no significant association between other dietary patterns and NAFLD.

**Table 5:** Multivariable analysis for the risk of NAFLD by tertile of dietary patterns

	T1 (lowest)	T2 (middle)		T3 (highest)	
		OR	95%CI	OR	95%CI
Healthy pattern					
n	93	94		94	
Crude	1.0	0.46*	0.25–0.87*	0.71	0.39–1.30
Model 1	1.0	0.47*	0.25–0.91*	0.76	0.41–1.40
Model 2	1.0	0.46*	0.23–0.92*	0.8	0.42–1.55
Western pattern					
n	94	94		93	
Crude	1.0	0.83	0.45–1.51	0.65	0.35–1.20
Model 1	1.0	0.92	0.49–1.71	0.71	0.38–1.34
Model 2	1.0	0.85	0.44–1.66	0.71	0.36–1.39
Snack pattern					
n	93	94		94	
Crude	1.0	0.77	0.41–1.43	1.03	0.56–1.89
Model 1	1.0	0.80	0.42–1.50	1.04	0.56–1.95
Model 2	1.0	0.84	0.42–1.66	1.13	0.58–2.20

Model 1: Adjusted for age, physical activity, smoking behavior

Model 2: Adjusted for Model 1 + BMI

Participants are categorized to low, medium and high adherence to each dietary pattern based on tertile of dietary pattern scores

*p < 0.05

Energy and nutrient intakes by tertile of healthy patterns are shown in Table 6. The group with the highest dietary pattern scores (T3) showed higher energy and nutrient intake than other groups.

**Table 6:** Comparison of energy and nutrient intakes by tertile of healthy patterns

Variables	T1 (lowest)	T2 (middle)	T3 (highest)	*P* value
Healthy pattern				
n	93	94	94	
Energy (kcal)	1434 (1204–1651)	1652 (1377–1892)	1883 (1681–2258)	< 0.001
Protein (g)	63.5 (57.2–69.6)	66.7 (61.2–73.2)	68.1 (63.2–74.9)	0.005
Total fat (g)	49.4 (43.2–56.9)	52.9 (43.6–60.4)	55.2 (49.3–61.3)	< 0.001
Carbohydrate (g)	238.2 (216.6–259.8)	236.0 (215.3–259.5)	239.2 (220.5–255.5)	0.785

Data are shown as median (IQR)

*P* values were obtained from Kruskal Wallis test

Participants are categorized to low, medium, and high adherence to each dietary pattern based on tertile of dietary pattern scores

Adjusted for energy by using residual method

## Discussion

Evidence of the association between dietary patterns and NAFLD in Japan is limited. In this study comprising adult Japanese men, we found three dietary patterns, namely, healthy, western, and snack patterns. The score of healthy dietary pattern, which included a high intake of vegetables, seaweeds, mushrooms, pulses, and potatoes and starches, was associated with a lower risk of NAFLD. To the best of our knowledge, this is the first study to evaluate associations between dietary pattern and NAFLD risk in Japanese men.

Our result corresponds to the previous findings that a dietary pattern consisting of high vegetable intake was associated with a decreased risk of NAFLD [9, 10, 20, 21]. The association between dietary pattern and NAFLD has been reported in various countries. In a Chinese study, the grains-vegetables pattern, characterized by high intake of vegetables, tubers, and mushrooms, was associated with a lower prevalence of NAFLD [21]. Similarly, in a Brazilian study [22] a healthy dietary pattern, composed of fruit, greens and vegetables, and margarine, was inversely associated with the prevalence of NAFLD. Furthermore, in a Greek study, there is a negative correlation between an unsaturated fatty acid dietary pattern and the risk of developing NAFLD [23]. A recent Korean study also reported that a simple meal pattern, characterized by a high intake of fruits, eggs, dairy products, and nuts, was inversely correlated with NAFLD [9]. Moreover, in that study, the traditional dietary pattern, characterized by a high intake of green vegetables, fish, mushrooms, fermented soybeans, and seaweed, was associated with an increased risk of NAFLD. This is in contrast to the findings of the current study and other previous studies. This could be because a traditional Korean diet features several kinds of high-salt foods such as kimchi and doenjang. Accordingly, another study of a Korean population reported that a high-salt dietary pattern was associated with an increased risk of NAFLD [24]. Therefore, high-salt intake may have a stronger effect on the risk of NAFLD than vegetable and mushroom intake. In present study, the group with the highest score of healthy pattern (T3) had high-salt intake, which could be the reason the risk of NAFLD was not attenuated in this group.

The NAFLD group in the current study consumed a low amount of mushrooms, and individuals in the lowest quartile of mushroom consumption was associated with a higher risk of NAFLD than individuals in the highest quartile after adjusting for age, physical activity, and BMI. Mushrooms contain many nutritional components such as dietary fibers, vitamins (e.g. vitamin D, niacin), minerals and polyphenols [25]. Bacteria that live in the intestines convert insoluble dietary fiber ferment to short chain fatty acids, such as butyrate. Short chain fatty acids play an important role in gluconeogenesis; therefore, they affect the development of NAFLD. A previous study demonstrated that butyrate may relieve inflammation and could be associated with lower prevalence of NAFLD [26]. Vitamin D has anti-inflammatory and anti-fibrotic effects [27]. Niacin inhibits fatty acid flux from adipose tissue to liver, reduces hepatic triglyceride synthesis and increases hepatic lipid oxidation [28]. Mushrooms provide antioxidants and anti-inflammatory agents, such as minerals, polyphenols, vitamins and polysaccharides [29, 30]. Those nutrients could protect from oxidative stress and inflammation, which are major risk factors of NAFLD [31, 32]. Animal experiments showed that mushrooms might have a protective benefit against NAFLD [33, 34]. Gil-Ramirez et al. reported that ergosterol-enriched extracts from mushrooms (doses were calculated taking into account the hypocholesterolemic minimal daily intake of plant sterols recommended by European Food Safety Authority) lowered hepatic triglyceride levels and modified the mRNA expression of cholesterol-related genes, thereby making them useful for limiting hepatic steatosis and preventing NAFLD [33]. Drori et al. also suggested that vitamin-D-enriched mushroom extracts were associated with significant attenuation of the rate of total body fat accumulation, along with a decrease in hepatic fat content, and had a protective effect against NAFLD [34]. Mushrooms contain vitamin D and insoluble dietary fiber abundantly. Collectively, these findings indicate that mushrooms may delay or prevent the development of NAFLD in humans also. A recent Chinese study has shown that higher mushroom intake was significantly associated with lower prevalence of NAFLD among Chinese adults [35]. This result is in agreement with our result. Further human researches are needed to validate these results.

Mushrooms (e.g., *Lentinula edodes*, *Flammulina velutipes*, and *Hypsizygus marmoreus*) are often used in the Japanese diet of Washoku in which they are often cooked along with other vegetables and seaweeds. High intake of mushrooms is considered an indicator of a healthy diet. The healthy pattern in the present study appears to resemble the ichiju-sansai (one soup and three side dishes) pattern of Washoku, suggesting that following the Washoku diet can help prevent NAFLD in Japanese men. However, the healthy pattern was associated with a decreased risk of NAFLD, and the effect was lost when a high amount of food was consumed regardless of whether it was a healthy diet. The risk of NAFLD was not attenuated in the highest tertile of food consumption within the healthy pattern, possibly because of the high energy and total fat intake (Table 6). This indicates that even a healthy diet should include consumption of food only in appropriate amounts.

Our study had some limitations. First, this study used a cross-sectional design. Thus, causal relations between dietary patterns and NAFLD could not be investigated. Long-term dietary data are needed to determine this relationship. Second, dietary, exercise and smoking behaviors were determined using self-reported questionnaires, which could be influenced by subjects’ biases, including under- and over-reporting. Third, we used only physical activity data for leisure-time. Participants’ physical activity for occupational work was unclear. Finally, NAFLD was diagnosed using ultrasonography instead of the gold standard method of liver biopsy. However, this ultrasonography is the optimal method for NAFLD diagnosis in the healthy population, as it is non-invasive. Despite these limitations, our study remains valuable, and to the best of our knowledge this is the first study to investigate the impact of dietary patterns on NAFLD among Japanese men. Further studies are needed to clarify our findings.

## Conclusions

The score of healthy dietary pattern composed of mushrooms, vegetables, and seaweeds was associated with a decreased risk of NAFLD in Japanese men. The components of healthy dietary pattern (seaweeds, vegetables, and soybean foods) are often used in Washoku. Therefore, we believe that this healthy pattern closely resembles the diet of Washoku, which indicates that Washoku may help prevent or delay NAFLD in Japanese men. Furthermore, dietary counseling which includes recommendations for appropriate salt consumption should be implemented.

## Data Availability

The datasets used and/or analyzed during the current study are available from the corresponding author on reasonable request.

## Abbreviations

ALT: Alanine aminotransferase
AST: Aspartate aminotransferase
BMI: Body mass index
CI: Confidence intervals
DBP: Diastolic blood pressure
FBS: Fasting blood sugar
FFQ: Food frequency questionnaire
γ-GTP: Gamma-glutamyl transpeptidase
HbA1c: Hemoglobin A1c
HDL-C: High-density lipoprotein cholesterol
IQR: Interquartile range
LDL-C: Low-density lipoprotein cholesterol
MET: Metabolic equivalent
NAFLD: Nonalcoholic fatty liver disease
OR: Odds ratio
SBP: Systolic blood pressure
T-CHO: Total cholesterol
TG: Triglycerides
